# Assessment of pH Value and Release of Calcium Ions in Calcium Silicate Cements: An In Vitro Comparative Study

**DOI:** 10.3390/ma16186213

**Published:** 2023-09-14

**Authors:** Rubén Herrera-Trinidad, Pedro Molinero-Mourelle, Manrique Fonseca, Adrian Roman Weber, Vicente Vera, María Luz Mena, Vicente Vera-González

**Affiliations:** 1 Department of Conservative Dentistry and Orofacial Prosthodontics, Faculty of Dentistry, Complutense University of Madrid, 28040 Madrid, Spain; 2 Department of Reconstructive Dentistry and Gerodontology, School of Dental Medicine, University of Bern, 3007 Bern, Switzerland; 3 Department of Prosthodontics and Operative Dentistry, School of Dental Medicine, Tufts University, Boston, MA 02111, USA; 4 Department of Analytics Chemistry, Faculty of Chemical Sciences, Complutense University of Madrid, 28040 Madrid, Spain

**Keywords:** biomaterial, ion release, pH, mineral trioxide aggregate, MTA, calcium-silicate-based cements

## Abstract

The goal of this study was to evaluate the pH and the release of calcium from four calcium-silicate-based cements. Methods: Four materials were tested (ProClinic MTA; Angelus MTA; ProRoot MTA; Biodentine). The palatal canal root of acrylic upper molars was filled with each cement. Afterwards, they were set in phosphate-buffered saline. Measurements were taken by atomic adsorption spectroscopy (AAS) at 3, 24, 72, 168, 336, 672, and 1008 h. The pH was measured at the same timepoints. Kruskal–Wallis tests were carried out in each period, as the Kolmogorov–Smirnov and Shapiro–Wilk tests showed no parametric results. Results: Significant differences (*p* < 0.05) in calcium release were found at the 3-, 24-, and 72-hour evaluations. All of the analyzed groups presented a release of calcium ions up to 168 h, and the general tendency was to increase up to 672 h, with a maximum release of 25.45 mg/g in the ProRoot group. We could only observe significant differences (*p* < 0.05) in pH value over 168 h between the Biodentine (7.93) and Angelus MTA (7.31) groups. Conclusions: There were significant differences (*p* < 0.05) in calcium release. Nevertheless, no significant differences (*p* > 0.05) in the pH values were found at the studied timepoints, except for the values at 168 h.

## 1. Introduction

Calcium silicate cements (CSCs) mostly comprise dicalcium silicate (Ca_2_SiO_4_) and tricalcium silicate (Ca_3_SiO_5_). These particles primarily release calcium hydroxide and calcium silicate hydrate gel via a hydration reaction [1]. The first historical record of the use of a calcium silicate cement in dentistry was in 1878, when the German dentist Dr. Witte used Portland cement to fill dental root canals [2]. However, it was not until 1993 that Torabinejad described mineral trioxide aggregate (MTA) [3]. Later, in 1995, it was patented after the addition of bismuth powder [4].

MTA is indicated for apical fillings, direct pulp capping, canal perforation repair, and apexification [5,6]. However, MTA has some limitations to its use, such as working time, difficulty to manipulate, discoloration, and high economic cost [7]. In order to overcome these limitations, Biodentine™ has been developed; this material is also based on calcium silicates [8]. Both mineral trioxide aggregate (MTA) and Biodentine™ are materials that are combinations of dicalcium and tricalcium silicates, together with metal ions [9,10].

Calcium and hydroxyl ions are the main chemical components released by calcium silicate cements in water [3]. The release of these ions is responsible for these materials having certain properties, among which are the differentiation of pulp cells, cementoblasts, osteoblasts, periodontal fibroblasts, mesenchymal stem cells, and hard-tissue mineralization [11,12,13,14,15]. Calcium silicate cements allow for the angiogenic differentiation of pulp cells. In fact, the ionic products of calcium silicates induce osteogenesis and angiogenesis [16].

Alkaline pH values accelerate apatite nucleation, because OH ions become soluble and can be included in apatite—an essential component of the tooth matrix. In addition, hydroxyl ions stimulate the release of alkaline phosphatase and bone morphogenetic protein 2 (BMP2), which are involved in mineralization processes [17]. These processes of mineralization of the hard tissue and formation of apatite are responsible for the sealing of these materials and, therefore, for their indications.

There are currently few studies comparing the pH and the release of calcium ions in the medium between different types of calcium silicate and bioceramic cements for periods longer than seven days. The release after this period is an important question, since the bone matrix crystallization occurs on the surface of the bioceramic material seven days after injury [18].

According to the study by Natale et al., all of the materials decrease their calcium release at 28 days [19]. Due to this, we carried out the last measurement of the variables at 42 days. Previous studies have shown similar results, like those obtained by Cavenago et al. [20], Gandolfi et al. [11], Kim et al. [21], Bernardi et al. [22], and Irawan et al. [18], who expressed the results with a heterogeneous unit of measurement, making comparison with other studies difficult. Considering this heterogeneity limitation, further studies are necessary to allow a direct comparison.

Therefore, the aim of the present study was to evaluate the release of calcium from four calcium-silicate-based cements, along with the pH reached, to determine which material presents the highest calcium release and pH changes. The null hypothesis was that there would be no differences in the release of calcium and the pH reached among the calcium-silicate-based cements.

## 2. Materials and Methods

### 2.1. Study Design

An in vitro comparative study was performed at the Department of Conservative Dentistry and Orofacial Prosthodontics based on the previously reported protocols of Cavenago et al. [20], Gandolfi et al. [17], and Irawan et al. [18], evaluating 40 standardized acrylic teeth (“Moulding Molar/Moulding Root” by PolyJet printing. Prototype: 0076209; AIJU, Ibi, Alicante, Spain) with root canals, and an apical stop was created with an 80 file (K-Flexofile ISO 80; DentsplyTulsa Dental Specialties, Tulsa, OK, USA; REF: A012D03108004). Since no human samples were used, ethical approval was not required from the Ethics Committee Research of Complutense University Hospitals for this in vitro study.

### 2.2. Study Setup and Specimen Fabrication

The teeth were randomly distributed into 4 groups (*n* = 10), in which the following materials were assessed: ProClinic MTA (ProClinic, Maruchi, Wonju-si, Republic of Korea), Angelus MTA (Angelus, Londrina, PR, Brasil), ProRoot MTA (DentsplyTulsa Dental Specialties, Tulsa, OK), and Biodentine (Septodont, Saint Maurde Fossés, France).

Acrylic teeth were weighed on a previously calibrated high-precision laboratory balance (Sartorius Secura^®^ Analytical Balance 220 g 0.1 mg. Item no.: SECURA224-1CEU). Afterwards, the instrumented canal was filled in its last three millimeters with the cement corresponding to the group, using an MTA transporter instrument (CHL MEDICAL SOLUTIONS, Srl., Milan, Italy; REF: 59814), and they were weighed again to control the amount of material introduced into the tooth. Subsequently, the teeth were placed individually in tubes with 10 mL of a buffered solution (phosphate-buffered saline, batch number 120438, Condalab, Madrid, Spain) that resembled the physiological medium [17]. In addition, four tubes were prepared as negative controls: two tubes containing one undrilled acrylic tooth, and two tubes without acrylic teeth. All of the included tubes were stored in a 37 °C distilled water bath throughout the experiment.

The calcium release determination was performed by atomic absorption spectroscopy (AAS) using an acetylene–air flame and equipped with a specific calcium-ion cathode lamp (Atomic Absorption Spectrophotometer Perkins Model 3100. PerkinElmer Inc., Waltham, MA, USA). An external calibration was performed in 25 mL flasks, and an additional control flask without calcium was prepared for the instrumental calibration. The absorbance was measured at 422.7 nm, and the calcium concentration was determined by interpolating its value in the previous calibration.

The pH determination was performed with a pH meter (Crison Basic 20+, CRISON Instruments, S.A., Alella, Barcelona, Spain) previously calibrated using buffer solutions of pH 4.7 and 9. Calcium ion release and pH measurements were performed at the following six timepoints on samples and controls: 24 h, 72 h, 168 h, 336 h, 672 h, and 1008 h.

SEM analysis using a JEOL JSM 6335F microscope and X-ray diffraction were carried out to determine the composition of each cement and to conduct a descriptive evaluation of the cements at 1008 h, taking a sample from each group.

### 2.3. Statistical Analysis

Data analysis was performed by using SPSS 28.0.1.0 statistical software (IBM Corp, Armonk, NY, USA). Kruskal–Wallis tests were carried out in each period, as the Kolmogorov–Smirnov and Shapiro–Wilk tests showed no parametric results. The level of significance was set to 0.05.

## 3. Results

The results showed an increase in calcium release and pH changes, especially over the first week (168 h). The pH values increased until reaching 7.93 in the Biodentine group at 168 h, which was the highest value in our study. In the rest of the groups the pH reached 7.48–7.51 at 24 h and then decreased.

**Figure 1:**
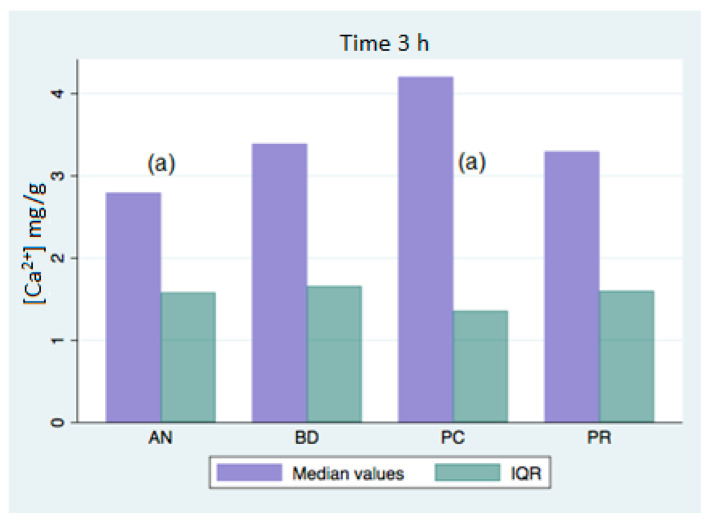
Released calcium ion concentration medians at 3 h, in mg/g; (a) shows significant differences between group pairs.

**Figure 2:**
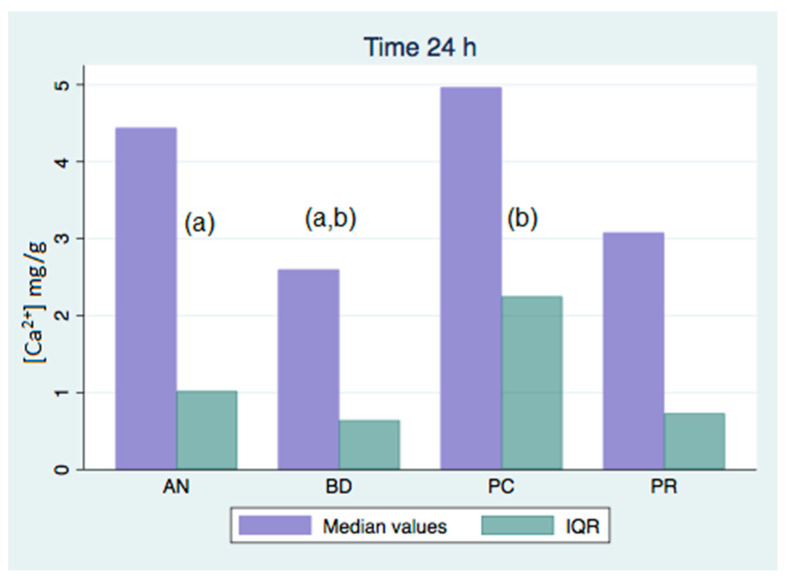
Released calcium ion concentration medians at 24 h, in mg/g; (a,b) show significant differences between group pairs.

**Figure 3:**
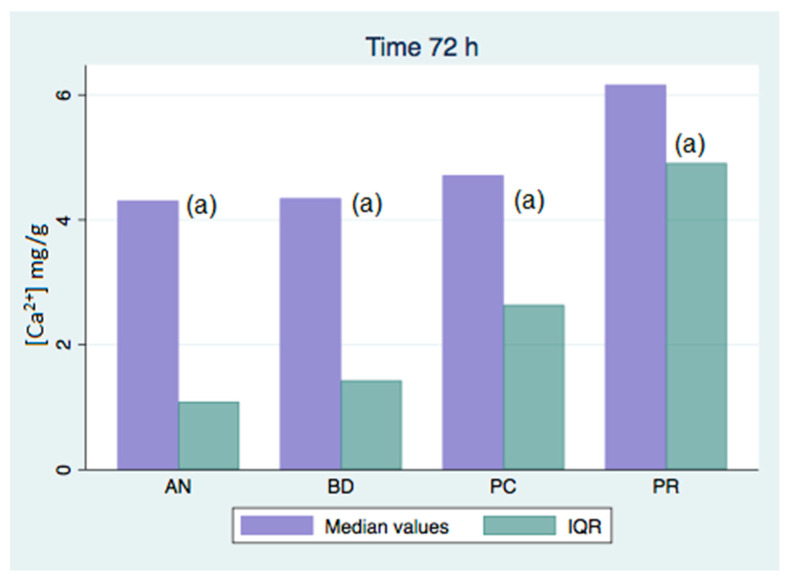
Released calcium ion concentration medians at 72 h, in mg/g; (a) shows significant differences between group pairs.

**Figure 4:**
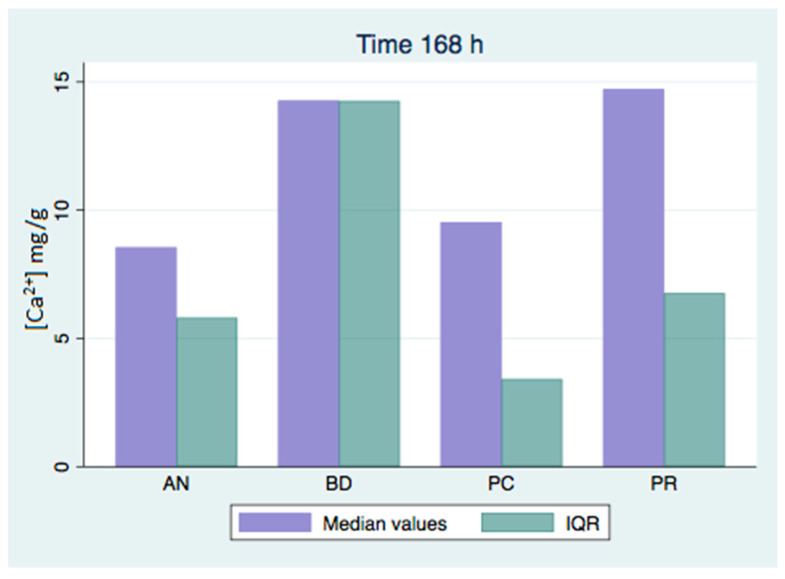
Released calcium ion concentration medians at 168 h, in mg/g.

**Figure 5:**
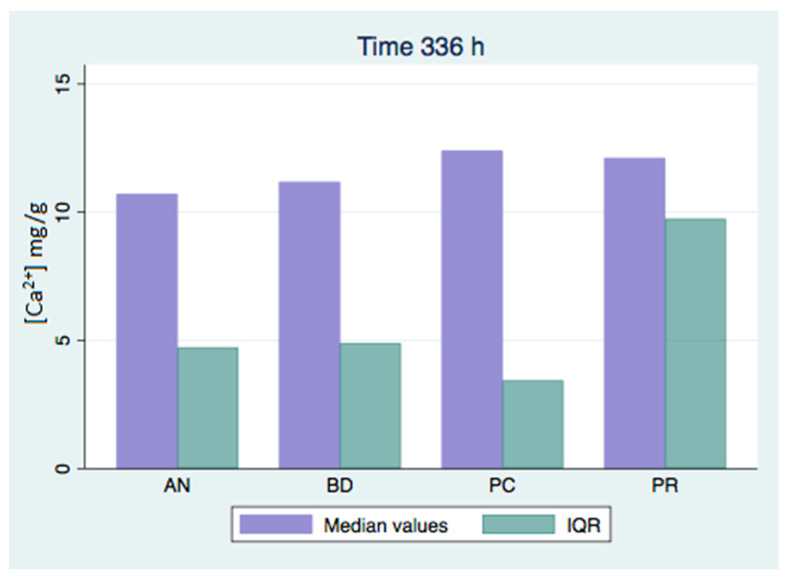
Released calcium ion concentration medians at 336 h, in mg/g.

**Figure 6:**
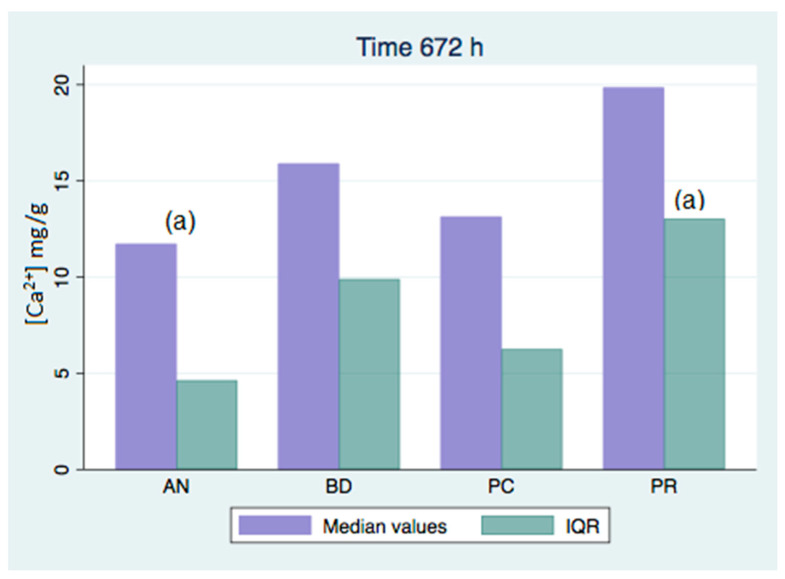
Released calcium ion concentration medians at 672 h, in mg/g; (a) show significant differences between group pairs.

**Figure 7:**
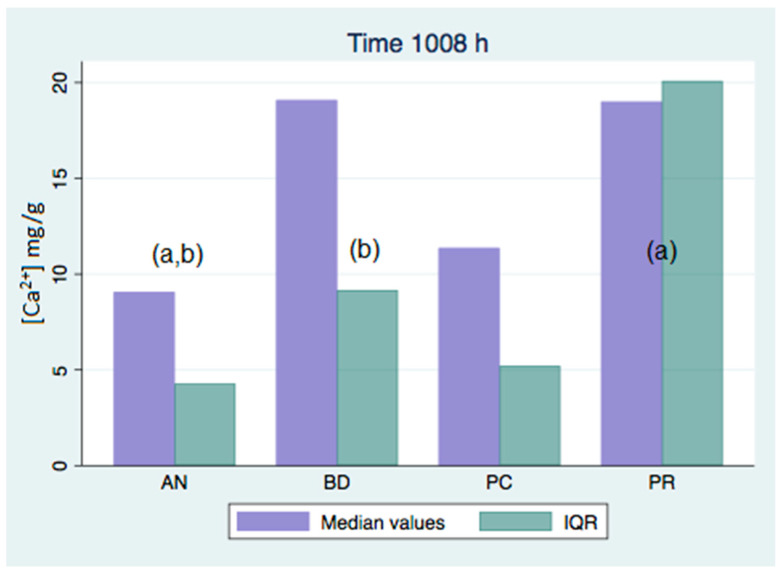
Released calcium ion concentration medians at 1008 h, in mg/g; (a,b) show significant differences between group pairs.

**Figure 8:**
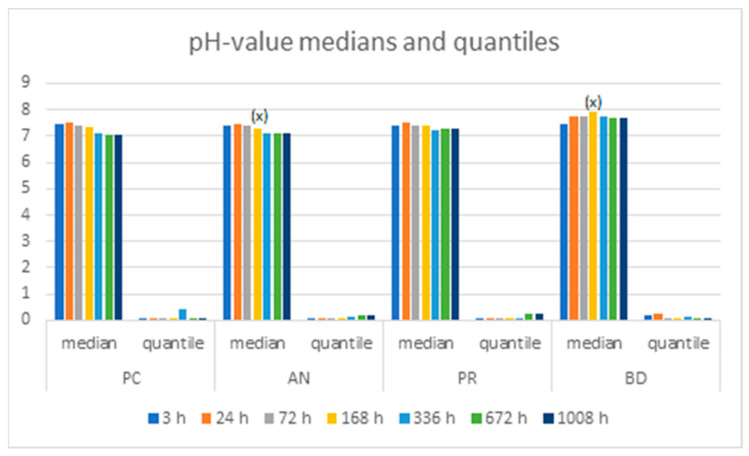
Medians and quantiles of pH over time; (x) shows significant differences between group pairs.

All of the analyzed groups presented a release of calcium ions up to 7 days (168 h), and the general tendency was to increase up to 28 days. By that time, the ProClinic and Angelus groups decreased their release of calcium ions, while the ProRoot and Biodentine groups continued to increase their ion release. The pH value increased until reaching 7.93 in the Biodentine group. Significant differences (*p* < 0.05) were noticed over 168 h between the BD (7.93) and AN (7.31) groups. Medians and quantiles were also calculated due to nonparametric data (Table 1 and Table 2; Figure 1, Figure 2, Figure 3, Figure 4, Figure 5, Figure 6, Figure 7 and Figure 8).

**Figure 9:**
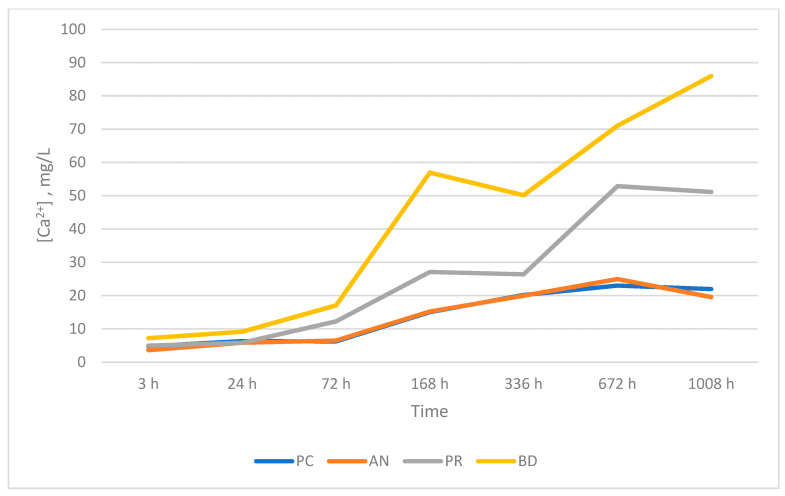
Mean released calcium ion concentrations, measured in mg/L.

At 3 h, there were significant differences (*p* < 0.05) in calcium release, where the ProClinic group reached 4.40 mg/g, compared to 2.95 mg/g in the group Angelus. Over 24 h, both the ProClinic group (with 5.37 mg/g) and the Angelus group (with 4.36 mg/g) had significant differences (*p* < 0.05) from the Biodentine group (with 2.61 mg/g). At 72 h, the ProClinic group released 4.71 mg/g, the Angelus group released 4.57 mg/g, the ProRoot group released 6.64 mg/g, and the Biodentine group released 4.28 mg/g. There were significant differences (*p* < 0.05) between the groups PC–PR, PC–BD, BD–AN, and AN–PR. All analyzed groups presented a release of calcium ions up to 168 h, and the general tendency was to increase up to 672 h, with a maximum release of 25.45 mg/g in the PR group. We observed a general increase in calcium release (in mg/L) in all of the groups, where the BD group kept increasing with a higher concentration than the other groups (Table 3; Figure 9). However, the data were not statistically analyzed.

**Figure 10:**
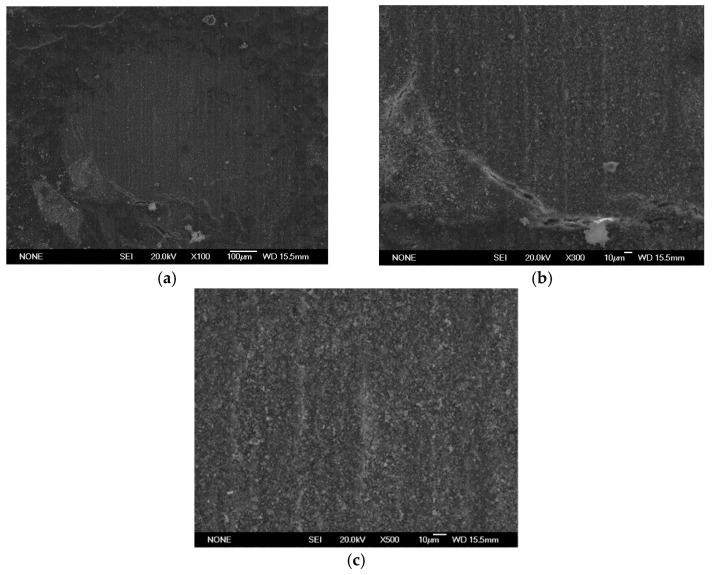
(**a**–**c**) Group PC sample viewed by SEM at ×100, ×300, and ×500, respectively, after 1008 h.

**Figure 11:**
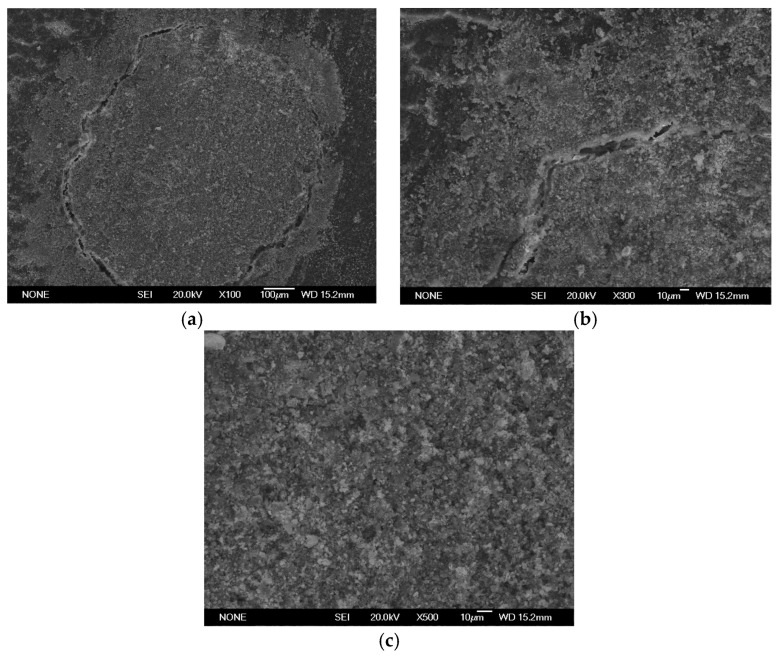
(**a**–**c**) Group AN sample viewed by SEM at ×100, ×300, and ×500, respectively, after 1008 h.

**Figure 12:**
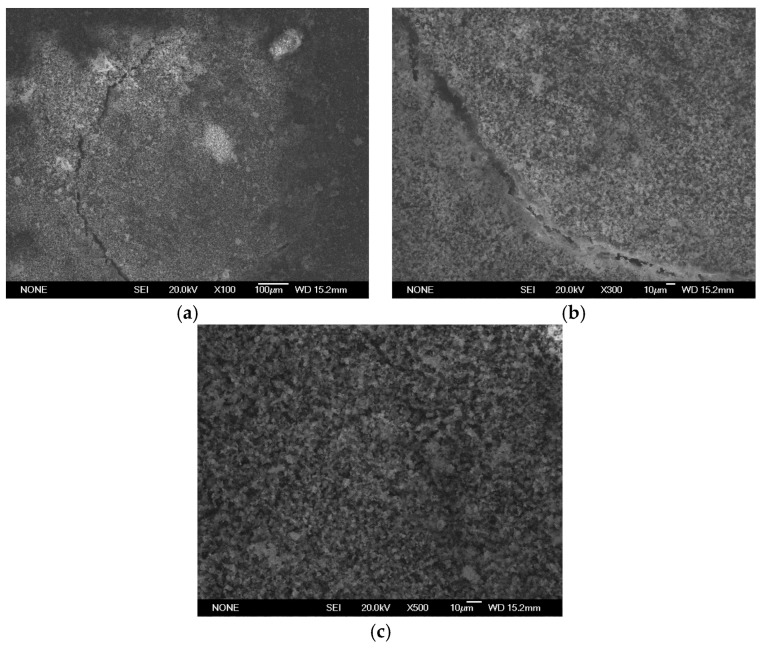
(**a**–**c**) Group PR sample viewed by SEM at ×100, ×300, and ×500, respectively, after 1008 h.

**Figure 13:**
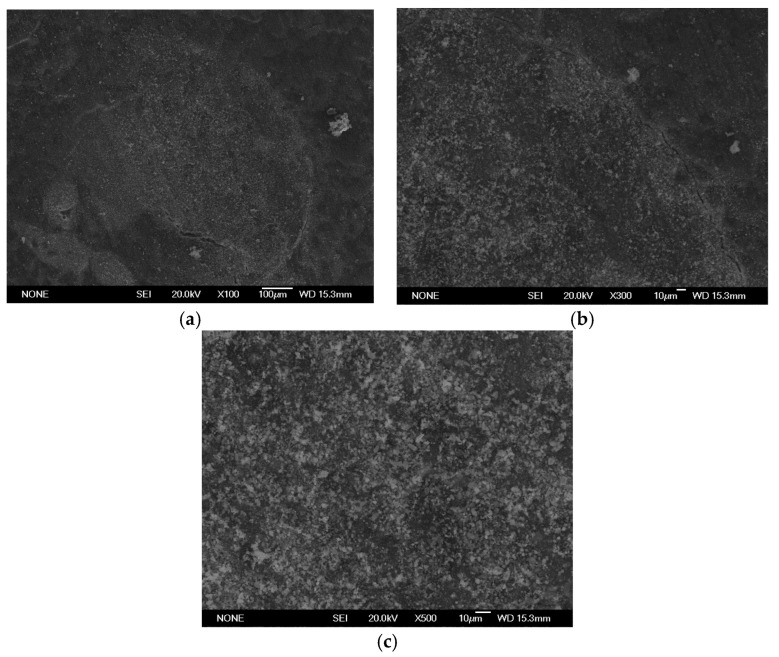
(**a**–**c**) Group BD sample viewed by SEM at ×100, ×300, and ×500, respectively, after 1008 h.

**Figure 14:**
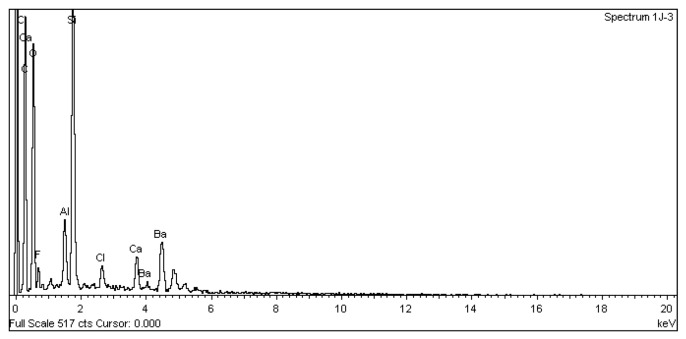
EDX spectrum from a PC sample.

**Figure 15:**
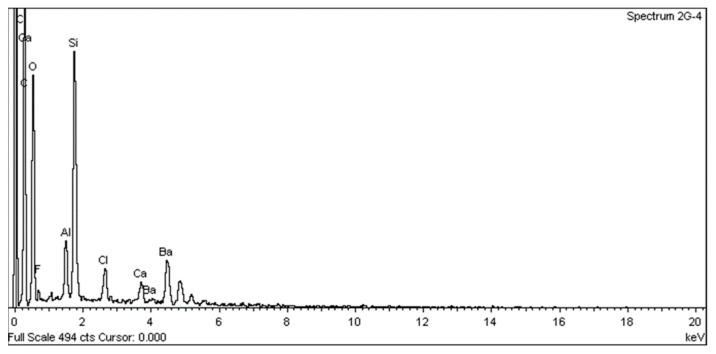
EDX spectrum from an AN sample.

**Figure 16:**
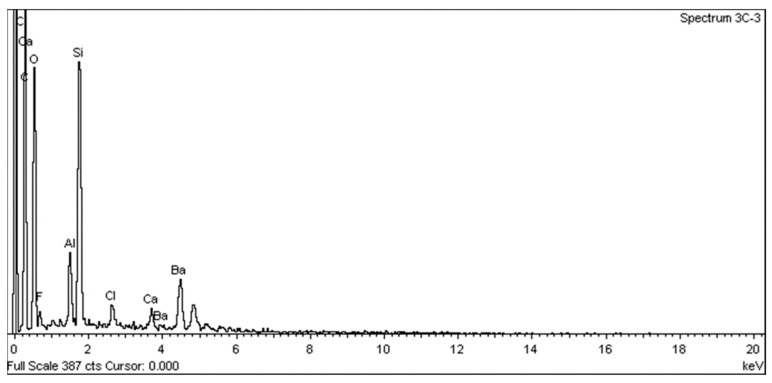
EDX spectrum from a PR sample.

**Figure 17:**
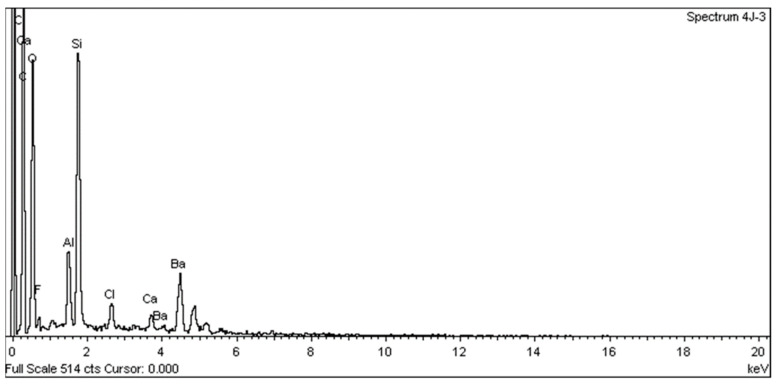
EDX spectrum from a BD sample.

**Figure 18:**
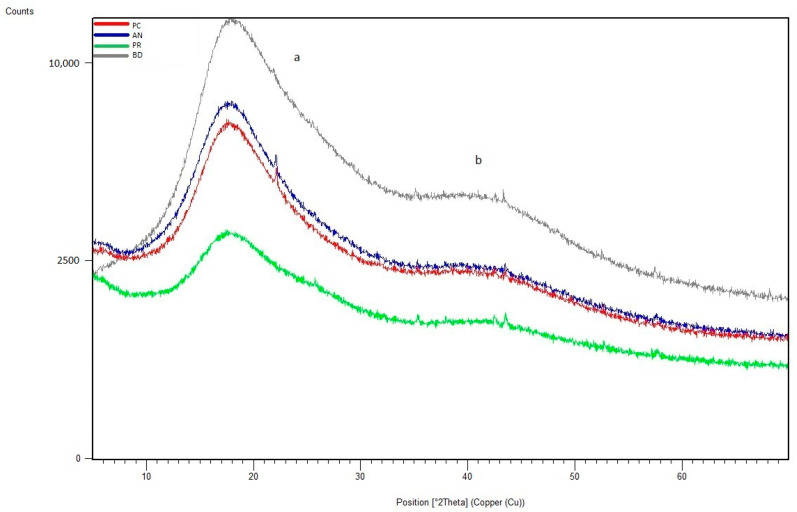
X-ray diffraction results, where different phases were identified as indicated (levels (a,b)).

SEM analysis using a JEOL JSM 6335F microscope and X-ray diffraction were carried out in order to determine the present phases and chemical composition of each cement. In this sense, a descriptive evaluation of the cements was conducted at 1008 h, taking a sample from each group. It was determined that all of the cements had similar chemical compositions, and they were identified as calcium aluminosilicates (CaAl_2_Si_6_O_16_) (Figure 10, Figure 11, Figure 12, Figure 13, Figure 14, Figure 15, Figure 16, Figure 17 and Figure 18).

## 4. Discussion

The results of the present study showed significant differences (*p* < 0.05) in the concentration of calcium release and pH changes at the studied times. According to the obtained results, the null hypothesis was rejected, since significant differences in the calcium release and pH values were detected between groups.

When the study sample is considered, a previous study by Irawan et al. [18] used 15 teeth per study group, while Cavenago et al, used 10 teeth [20]; in this sense, the present study was consistent with them. The analyses at 3, 24, 72, and 168 h were compared with the related literature, such as the study by Cavenago et al. We extended the analysis to 336 h, 672 h, and 1008 h to obtain comparative data among the various similar studies.

Evaluating the comparisons of the samples’ calcium ion release (mg/g), it was considered that the amount of material in the duct, despite the standardization of the samples, could be different. However, the present study analyzed the concentrations in mg/L to compare them with the study of Cavenago et al. [20]. Gandolfi et al. and Irawan et al. obtained the results of the release of calcium ions in ppm [17,18].

In the present study, the pH was calibrated at 4, 7, and 9, instead of at 4, 7, and 14 as previously reported by Cavenago et al.; in this way, the calibrations were easier to perform, since this method contributes to less-dispersed data. The pH data varied little over time and remained constant in all of the included groups. This fact could be due to the fact that the solution used was a buffered medium [23].

Other factors to consider that were not studied here could include the setting time of each material, along with its porosity, since these contribute to its subsequent consistency [24]. Another factor to consider is the oral environment. It should be noted that saliva lipids determine the buffering and antibacterial capacity of the environment, which could influence the pH variations occurring in the medium of calcium silicate cements [25].

According to Palczewska-Komsa et al. [26] the main components of MTA are CaWO_4_, Ca_3_SiO_5_, and Ca_2_SiO_4_ as the main phases of the composition. The content of calcium aluminate improves the biological response of HP MTA. In several scientific studies, the chemical composition of HP MTA was determined using energy-dispersive X-rays. Jiménez-Sánchez et al. [27] found that the structure of the HP MTA tricalcium silicate particles ensures a very close contact between the calcium silicate and calcium aluminate and, thus, favors the hydration reaction. However, according to our records, no W was found. This seems to correlate with the findings of Rochas et al. and Ertas et al., where no W was found in their composition studies of these materials [28,29].

We analyzed the release of calcium ions over time in the groups studied. In our first data record, at 3 h, the release of calcium ions was already observed, consistent with the findings of Gandolfi et al. However, according to the study of Gandolfi et al., it was not until 5 h when apatite formation began. This formation would reach a uniform thickness after 7 days [18].

Significant differences (*p* < 0.05) were observed in the comparisons at 3 h between the AN and PC groups, with the highest value being in the PC group. At 24 h, the PC group showed significant differences again, that time with respect to the BD group. Likewise, there were significant differences between the AN and BD groups, with the AN group being the one that presented a greater release of calcium ions at that point.

At 72 h, there were significant differences (*p* < 0.05) between all groups. This situation was only repeated at 42 days with respect to the AN group. At 28 days (672 h), there were significant differences, where the PR group presented higher figures than the AN group. At 1008 h, the BD group showed significant differences with respect to the AN group. We could see how the behavior of the different cements varied over time, not being comparable between any of the groups. If we compared the variations of our study (in mg/L) to those found in the study of Cavenago et al., we could not establish similarities. It is possible that this was due to the different environments in which the samples were studied [20].

We analyzed the pH values in comparison to similar studies. The pH values increased until reaching 7.93 in the Biodentine group at 7 days. Nevertheless, in the other three groups, the pH value increased up to 24 h and then decreased. The obtained values were consistent with those described by Cavenago et al., although the medium was ultrapure water. The values in the referenced studies could have been higher due to the included medium, in which the samples were not buffered and, therefore, the variation of the ions affected the pH to a greater extent [23].

The results of the study by Ceci et al. showed MTA to have greater pH values than Biodentine. Moreover, the values were higher in their study. The highest values were found for ProRoot MTA, reaching 12.48 at 3 h and 11.56 at 24 h. For all materials tested, a nonsignificant reduction in pH value was recorded after 24 h. [30]. In our study, the same situation seemed to arise for all of the MTA groups after 24 h. The pH results of Ceci et al. were consistent with those of Herrera-Trinidad, who carried out a similar study in an unbuffered medium [31], with ultrapure or deionized water. The pH values in our current study were never below 7.05. However, the pH values could have been lower due to the buffered medium. The buffered solution, according to the distributor, has a pH of 7.2 ± 0.1 at 25 °C. The pH is determined by the amount of H^+^ or OH^−^ ions that is needed until the pH value of a solution changes by 1. Therefore, a high release of H^+^/OH^−^ ions is needed to modify the pH in the medium. According to the study by Irawan et al., bioceramics release more calcium ions and reach higher pH levels than MTA and can increase their tissue regeneration capacity [18]. Our study obtained similar results, with the pH values in Biodentine™ being overall higher than in the other groups. However, there were significant differences in pH at 168 h between Biodentine™ and the MTA groups.

According to Kim et al., due to this alkaline pH, calcium-silicate-based materials can be bactericidal, like calcium hydroxide; nevertheless, they can also induce necrosis of the root surface cells [21]. Another study by De Deus et al., using calcium silicate cements, presented an initial cytotoxic effect, which may have been due to the pH reached on the surface of the cements, which caused the denaturation of adjacent cells and medium proteins [32,33]. This may be related to the slight decreases in pH in the medium observed in the different studies.

Biodentine™ and MTAs have been reported to increase the release of TGF-β1, a modulator of tissue repair and mineralization [34,35], as well as the activation of kinases involved in odontoblastic differentiation [36,37] and cytokines that promote the inflammatory response [38,39]. The study by Machado et al. concurs on the cell proliferation capacity of calcium silicate cements, which, in this case, was significantly greater than in samples with composite [40]. Studies such as that by Benneti et al. showed biocompatibility and mineralization capacity in vivo [41].

According to Rajasekharan et al., each clinical application requires an adequate volume of calcium silicate cement at the repair site, depending on the severity. Furthermore, the area of biomaterial exposed to oral tissues varies widely. Despite the various studies analyzing pH and calcium release, there is no evidence of the environmental effects of pH, volume (Vol), and exposed area on the release of calcium and hydroxyl ions [42].

In addition, it is important to remember that the concentrations found in the solutions do not represent those that reach the enamel or dentin lesion, which would require more complex microanalytical techniques [43]. The beginning of calcium ion release and its implications for apatite formation are important, since, according to Sarkar et al., the biocompatibility, sealing capacity, and dentinogenic activity of MTA stem from the physicochemical reactions between MTA and tissues during hydroxyapatite formation [44].

Hard tissue can form next to MTA due to its alkalinity, sealing ability, and bioactivity [45]. These properties depend on the calcium ion release and pH. These variables were analyzed in our study. It is important to understand how different materials interact with our dental tissues and cells. As reported by Kim et al. [46], stem cells from human exfoliated deciduous teeth and human dental pulp stem cells had significantly low ALP activity after exposure to each material compared with the controls (i.e., cells cultured with osteogenic media). An elevated level of calcium release was found in all calcium silicate cements.

Further studies on natural teeth or in vivo would be necessary to assess variables related to the tooth and/or the environment.

## 5. Conclusions

Considering the limitations of the present in vitro study, the following conclusions can be drawn:

- There were significant differences in the concentration of calcium release between the different groups studied, with no group being predominant.
- There were no significant differences in the pH values at the timepoints studied, except for the values at 168 h. The pH values showed small variations throughout the evaluated period due to the buffered medium in which the samples were found.

## Figures and Tables

**Table 1:** Means and standard deviations of released calcium ion concentrations (mg/g) and pH values over time; (a,b,x) show significant differences between group pairs.

	ProClinic Group (PC) *n* = 10		Angelus Group (AN) *n* = 10		ProRoot Group (PR) *n* = 10		Biodentine Group (BD) *n* = 10	
	[Ca^2+^] mg/g	pH	[Ca^2+^] mg/g	pH	[Ca^2+^] mg/g	pH	[Ca^2+^] mg/g	pH
3 h	4.44 ± 1.08 (a)	7.43 ± 0.01	2.95 ± 0.82 (a)	7.41 ± 0.01	3.36 ± 1.19	7.41 ± 0.02	3.36 ± 1.22	7.48 ± 0.12
24 h	5.37 ± 1.35 (b)	7.51 ± 0.01	4.36 ± 0.81 (a)	7.48 ± 0.01	3.66 ± 1.64	7.50 ± 0.03	2.61 ± 0.37 (a,b)	7.75 ± 0.19
72 h	4.71 ± 1.22 (a)	7.39 ± 0.04	4.57 ± 1.91 (a)	7.38 ± 0.02	6.64 ± 2.89 (a)	7.40 ± 0.04	4.28 ± 0.99 (a)	7.73 ± 0.04
168 h	10.28 ± 3.17	7.35 ± 0.07	9.15 ± 3.80	7.31 ± 0.02 (x)	13.48 ± 5.74	7.39 ± 0.07	14.80 ± 9.03	7.93 ± 0.02 (x)
336 h	13.11 ± 5.10	7.12 ± 0.02	11.03 ± 2.49	7.13 ± 0.09	13.11 ± 6.13	7.22 ± 0.03	12.86 ± 6.32	7.76 ± 0.09
672 h	13.73 ± 3.97	7.05 ± 0.01	12.52 ± 3.92 (a)	7.10 ± 0.14	25.45 ± 18.63 (a)	7.28 ± 0.16	17.58 ± 6.99	7.67 ± 0.03
1008 h	12.31 ± 3.64	7.05 ± 0.01	9.35 ± 2.23 (a,b)	7.10 ± 0.14	23.83 ± 12.45 (a)	7.28 ± 0.16	20.07 ± 5.41 (b)	7.67 ± 0.03

Note: a, b, x; denote a subset of groups whose column proportions do not differ significantly from one another at the 0.05 level, with Bonferroni’s correction.

**Table 2:** Medians and quantiles of released ion calcium concentrations (mg/g) and pH values over time; (a,b,x) show significant differences between group pairs.

	ProClinic Group (PC) *n* = 10		Angelus Group (AN) *n* = 10		ProRoot Group (PR) *n* = 10		Biodentine Group (BD) *n* = 10	
	[Ca^2+^] mg/g	pH	[Ca^2+^] mg/g	pH	[Ca^2+^] mg/g	pH	[Ca^2+^] mg/g	pH
3 h	4.20 (1.44) (a)	7.43 (0.02)	2.79 (1.66) (a)	7.41 (0.01)	3.29 (1.92)	7.41 (0.03)	3.39 (1.69)	7.48 (0.17)
24 h	4.96 (2.50) (b)	7.51 (0.01)	4.44 (1.23) (a)	7.48 (0.01)	3.08 (1.44)	7.5 (0.05)	2.60 (0.67) (a,b)	7.75 (0.27)
72 h	4.71 (2.66) (a)	7.39 (0.06)	4.31 (1.44) (a)	7.38 (0.03)	6.16 (5.31) (a)	7.4 (0.06)	4.34 (1.67) (a)	7.73 (0.06)
168 h	9.54 (3.62)	7.35 (0.10)	8.55 (5.99)	7.31 (0.03)	14.72 (7.10)	7.39 (0.10)	14.27 (16.10)	7.93 (0.03) (x)
336 h	12.40 (5.68)	7.12 (0.04)	10.71 (4.88)	7.13 (0.14)	12.12 (10.72)	7.22 (0.05)	11.18 (5.29)	7.76 (0.13)
672 h	13.15 (7.05)	7.05 (0.01)	11.73 (4.84) (a)	7.1 (0.21)	19.85 (14.91) (a)	7.28 (0.23)	15.91 (10.73)	7.67 (0.05)
1008 h	11.37 (3.64)	7.05 (0.01)	9.07 (4.43) (a,b)	7.1 (0.21)	19.00 (21.85) (a)	7.28 (0.23)	19.09 (10.10) (b)	7.67 (0.05)

Note: a, b, x; denote a subset of groups whose column proportions do not differ significantly from one another at the 0.05 level, with Bonferroni’s correction.

**Table 3:** Mean released calcium ion concentrations, measured in mg/L.

[Ca^2+^], mg/L				
	ProClinic Group (PC)	Angelus Group (AN)	ProRoot Group (PR)	Biodentine Group (BD)
3 h	4.76	3.61	4.98	7.20
24 h	6.34	5.82	5.84	9.15
72 h	6.22	6.52	12.24	17.06
168 h	15.01	15.23	27.10	56.95
336 h	20.16	19.96	26.39	50.15
672 h	23.00	24.95	52.87	71.00
1008 h	21.94	19.57	51.13	85.95

## Data Availability

The data that support the findings of this study are partially available upon request from the corresponding author.
